# Supplementary figures and images for: Tumor Mutation Burden, Expressed Neoantigens and the Immune Microenvironment in Diffuse Gliomas
Source: Cancers (Basel). 2021 Dec 3;13(23):6092. doi: 10.3390/cancers13236092 (PMC8657099; doi:10.3390/cancers13236092)

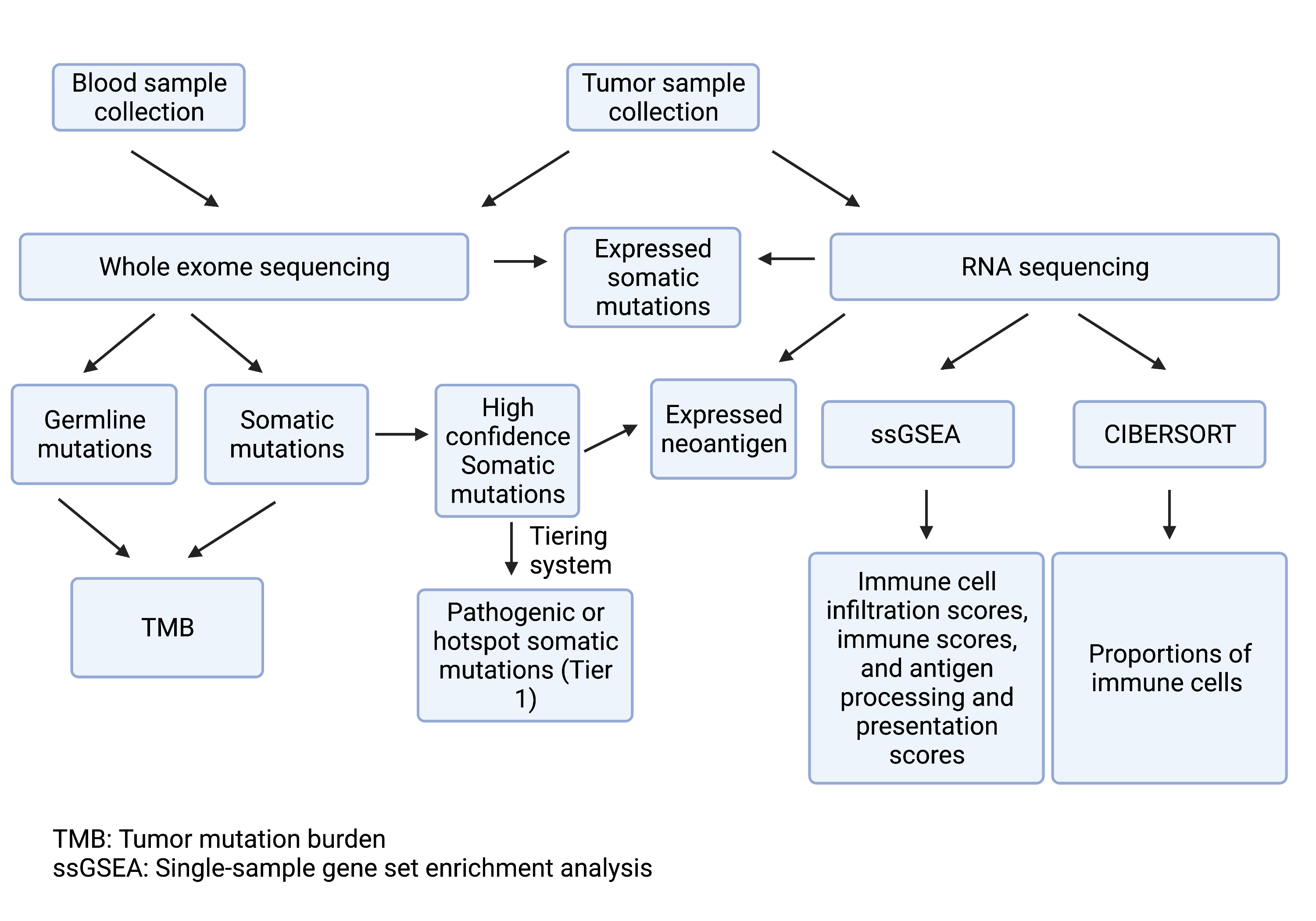

Supplement: Supplementary file 1 [file cancers-13-06092-s001.zip › cancers-1467068-supple/Figure S6.png]
